# Supplementary material for: Atacama Large Aperture Submillimeter Telescope (AtLAST) science: Probing the transient and time-variable sky
Source: Open Res Eur. 2025 Jan 14;4:132. Originally published 2024 Jun 28. [Version 2] doi: 10.12688/openreseurope.17686.2 (PMC11754950; doi:10.12688/openreseurope.17686.2)
Supplement: Peer review history

# Reviewer report: Reviewer response for version 2

**DOI:** 10.21956/openreseurope.20600.r49762

**Published:** 2025-01-29

**Authors:** Octavi Fors

**Affiliations:** 1 Institut de Ciencies del Cosmos, Universitat de Barcelona (Ringgold ID: 553397), Barcelona, Barcelona, Spain

This peer review report addresses the paper titled "Atacama Large Aperture Submillimeter Telescope (AtLAST) Science: Probing the Transient and Time-Variable Sky," submitted to Open Research Europe.

 This manuscript extensively discusses time-domain astronomy science cases that AtLAST can address. It also addresses the technical and observational requirements for meeting these science drivers.

 AtLAST will fill a niche in time-domain submillimeter astronomy, allowing deep and rapid follow-up observations triggered from other wavelengths.

 This manuscript deserves to be accepted, but the following minor comments and typos should be considered.

**General comments:**

 As mentioned in the last sentence of the second paragraph of Sect.1.3, Argus Array will be an optical all-sky, high-duty cycle survey in excellent synergy with AtLAST. I suggest citing this facility as Law et al. (2022).

 I also suggest mentioning Evryscope systems as precursors of Argus and citing these in Law et al. 2015).

 The authors highlight that submillimetric density flux from stellar flares have durations between few seconds and minutes. Citing MacGregor et al. (2021) would help to exemplify the <10s extremely short duration of Proxima Cen superflare as observed with ALMA.

 The authors point out in Sect.2.2.2 the potential of simultaneous multi-wavelength observations of stellar flares, including with AtLAST. The near future UV time-domain astronomy, where flares are especially prominent for main sequence late-type stars, may be mentioned as one of the earliest triggering observations.

 Wide FoV satellites such as ULTRASAT and the increasing number of CubeSats covering large areas of sky with NUV and/or FUV sensors will soon change the current panorama of UV time-domain astronomy.

 Even though the expected pointing accuracy is reported in Mroczkowski et al.2025, it may be good to explicitly mention in this manuscript. Stars generally form in cluster environments, and hence single-dish observations could be confusing. This is important to ambiguously discern the emitting source. 

 The manuscript focuses on the continuum variability and there is no mention of the variability of spectral lines. For instance, CH3OH maser emission is found to be highly variable in high-mass protostars. I understand that adding the spectral line science cases will drastically change the scope and length of the manuscript. My suggestion would be to highlight that the present manuscript focuses on the continuum emission and leaves the variability of spectral lines for a forthcoming work. In this sense, it would be good to clarify in Table 1 that the sensitivity reported corresponds to continuum bands.

**Minor comments and typos:**

 Plain language summary: I would add “A wide range of ASTROPHYSICAL objects and processes observable…”

 P5: please include Atacama Large Millimeter/submillimeter Array (ALMA) when first introduced. In the same page, the acronym SO LAT needs to be fully written. 

 P5.Sect.1.1. Please unify language in several instances of the manuscript. For example: "star-forming regions" and "star forming regions", "Galactic center" and "Galactic Center", "multi-chroic" and "multichroic", "Galactic plane" and "Galactic Plane".

 P6: Add the acronym of Northern Extended Millimeter Array (NOEMA)

 P6: I would report the frequency range of MUSTANG-2: from 75 GHz to 105 GHz.

 P6:  when comparing the sensitivity of SO LAT and CCAT to AtLAST it would be good to state by what factor AtLAST will be more sensitive. 

 P7: SED needs to be introduced.

 Table 2: Missing parentheses in some units listed in the Characteristics column. In addition, I recommend specifying the ALMA configuration used to report its characteristics since the angular resolution depends on the configuration used.

 Please review missing spaces and hyphens, for example: "followingup", "wellsuited", “gammarays", etc.

 P6. Sect.1.1. The "Large Milimeter Telescope (LMT)" acronym should be defined the first time this is mentioned in the text, not the second. Also missing "l" in "Milimeter".

 P6. Sect.1.1. The acronym TNOs is defined more than once. Please review.

 P6. Sect.1.1. Missing "s" in "Still, targeted observation are".

 P8. Sect.2. Extra "t" in "targetted observations".

 P10. Sect.2.1.1. There is a missing "of" in "the polar region the asteroid".

 P17. Sect.2.3.3.The X-axis of Figure 5 has no label.

 P19. Sect.3.1. Uniformize "revolutionize" versus "revolutionise".

Is the study design appropriate and does the work have academic merit?

Yes

Is the work clearly and accurately presented and does it cite the current literature?

Yes

If applicable, is the statistical analysis and its interpretation appropriate?

Not applicable

Are all the source data underlying the results available to ensure full reproducibility?

Yes

Are the conclusions drawn adequately supported by the results?

Yes

Are sufficient details of methods and analysis provided to allow replication by others?

Yes

Reviewer Expertise:

Reviewer Expertise: Time-domain astronomy, multiscale and multi-messenger stellar variability(flares), planetary habitability, high-angular resolution, wide field of view robotic astronomy.

I confirm that I have read this submission and believe that I have an appropriate level of expertise to confirm that it is of an acceptable scientific standard.

## References

[1] : Evryscope Science: Exploring the Potential of All-Sky Gigapixel-Scale Telescopes. *Publications of the Astronomical Society of the Pacific* .2015;127(949) : 10.1086/680521 234-249 10.1086/680521

[2] : Discovery of an Extremely Short Duration Flare from Proxima Centauri Using Millimeter through Far-ultraviolet Observations. *The Astrophysical Journal Letters* .2021;911(2) : 10.3847/2041-8213/abf14c 10.3847/2041-8213/abf14c

[3] : Low-cost Access to the Deep, High-cadence Sky: the Argus Optical Array. *Publications of the Astronomical Society of the Pacific* .2022;134(1033) : 10.1088/1538-3873/ac4811 10.1088/1538-3873/ac4811

# Reviewer report: Reviewer response for version 2

**DOI:** 10.21956/openreseurope.20600.r49729

**Published:** 2025-01-22

**Authors:** Yuhan Yao

**Affiliations:** 1 University of California Berkeley, Berkeley, California, USA

The authors have addressed my comments. I recommend the indexing of this article.

Is the study design appropriate and does the work have academic merit?

Yes

Is the work clearly and accurately presented and does it cite the current literature?

Yes

If applicable, is the statistical analysis and its interpretation appropriate?

Not applicable

Are all the source data underlying the results available to ensure full reproducibility?

Yes

Are the conclusions drawn adequately supported by the results?

Yes

Are sufficient details of methods and analysis provided to allow replication by others?

Yes

Reviewer Expertise:

time domain astronomy; high energy astrophysics

I confirm that I have read this submission and believe that I have an appropriate level of expertise to confirm that it is of an acceptable scientific standard.

# Reviewer report: Reviewer response for version 1

**DOI:** 10.21956/openreseurope.19113.r43443

**Published:** 2024-09-27

**Authors:** Kazumi Kashiyama

**Affiliations:** 1 Tohoku University, Sendai, Japan

This peer review report addresses the paper titled “Atacama Large Aperture Submillimeter Telescope (AtLAST) Science: Probing the Transient and Time-Variable Sky,” submitted to Open Research Europe.

 The authors discuss potential science cases for AtLAST, focusing on time-variable sources, and outline the technical requirements, observational strategies, and configurations necessary to optimize scientific output. The paper offers valuable insights for both experts and non-experts in the field, highlighting AtLAST’s capabilities and inspiring new avenues for future scientific exploration.

 Below, I have provided several minor comments that I believe will help improve the manuscript.

**3rd Paragraph, Page 6:**

 The abbreviation “Trans-Neptunian Objects (TNOs)” should be introduced when it first appears in the main text, which is in the 2nd paragraph on Page 6.

**Figure 1:**

 The blue and green points in the zoom-in box appear inconsistent with those in the zoom-out view.

**Sec. 2.2.3, Page 11:**

 The authors discuss novae in the section titled “Red Novae.” However, since the progenitor systems and the physical processes behind the emissions are different, I suggest discussing them in separate sections.

**Figure 2:**

 Figure 2 is not referenced in the main text. It would be better to refer to it, along with Tetarenko et al., 2021, in the first paragraph on Page 13.

**Sec. 2.3.3, Pages 15-16:**

 Tidal disruption events (TDEs) are mentioned several times in the manuscript but are not actually discussed. Since TDEs are also important and promising targets for AtLAST, I suggest including a discussion in this section. You could cover their emission properties, physical mechanisms, what is known and unknown about them, and potential observational strategies and scientific outcomes using AtLAST.

**Page 16 and Page 17**:

 The subsection 2.4 and 2.5 should be 2.3.4 and 2.3.5?

**1st Paragraph of Sec. 4.1, Page 18:**

 Providing an explanation of why “the exposure time needed will scale as Omega d_max^4” would be helpful for non-experts.

**“Coordinated observations”, Page 19**:

 “gammarays” <— typo

Is the study design appropriate and does the work have academic merit?

Yes

Is the work clearly and accurately presented and does it cite the current literature?

Yes

If applicable, is the statistical analysis and its interpretation appropriate?

Yes

Are all the source data underlying the results available to ensure full reproducibility?

Partly

Are the conclusions drawn adequately supported by the results?

Yes

Are sufficient details of methods and analysis provided to allow replication by others?

Partly

Reviewer Expertise:

high energy astrophysics, time-domain astronomy, multi-messenger astronomy

I confirm that I have read this submission and believe that I have an appropriate level of expertise to confirm that it is of an acceptable scientific standard, however I have significant reservations, as outlined above.

# Reviewer report: Reviewer response for version 1

**DOI:** 10.21956/openreseurope.19113.r42830

**Published:** 2024-09-09

**Authors:** Youdong Hu

**Affiliations:** 1 Instituto de Astrofìsica de Andalucìa (IAA-CSIC), Granada, E-18008, Spain; 2 Osservatorio Astronomico di Brera, Via E. Bianchi 46, Merate (LC), Lombardy, 2380, Italy

This paper provides a detailed discussion of the Atacama Large Aperture Submillimeter Telescope's promising vision and potential scientific outputs in the field of transient sources, mainly focusing on the millimeter and submillimeter wavebands. It also presents theoretical predictions for maximizing scientific outcomes regarding observational instruments and strategies. In the current era of multi-wavelength astronomy, this telescope will effectively fill the existing gaps in information in the millimeter to submillimeter bands. I believe this paper is worthy of indexing, but the following issues should be considered.

**Major comments:**

 1. Detection capability. The paper repeatedly mentions the wide FOV, multi-choric observation capabilities, and the advantages of these capabilities in different scientific contexts. However, it does not provide a detailed list of the telescope's key parameters. Additionally, while the text mentions advantages over other telescopes, it does not offer a comprehensive comparison. For example, it does not clearly demonstrate its indispensability compared to current instruments, like ALMA/ACT or others. Maybe including a table comparing the key parameters of this telescope with those of similar telescopes, along with a discussion of these comparisons, would help readers more intuitively understand the advantages of this telescope's detection capabilities.

 2. Fast radio burst and other transients. As a significant transient source discovered in recent years, the paper does not provide any information on the potential contributions of this telescope in this field. Additionally, while the paper mentions neutrino once and notes that the telescope will respond to Ice Cube alerts, there is a lack of further discussion on this topic.

 3. Timeline. Although this may not be the main focus of this paper, a brief construction timeline would be of great interest to the readers.

 4. Observation schedule. The paper includes a separate section introducing different types of observations, but it does not clarify the proportion of each type of observation or how they are scheduled. Specifically, regarding Too observations, the paper mentions that there will be an RRM mode to respond to automatic triggers, but it also states that semi-automatic triggers are accepted from proposals. However, it does not explain how conflicts between these two types of triggers (or different automatic triggers) would be resolved if they occur at same time.

 5. Subtitle for section 5. It looks just conclude the section 3 and section 4 which just take 2 pages for the whole paper, better change another one.

**Minors comments or typos:**

 1. P5. "previously known variable objects" might be confusing, as it can be easily mistaken for objects historically detected or first observed through other messenger.

 2. P5. "SO LAT". The citation for Simons Observatory better include in the brackets of SO LAT. In the paper, sometimes use "SO LAT" sometimes use "SO".

 3. P5. Cosmic Microwave Background telescope need citation.

 4. Abbreviations are missing full definitions upon their first appearance. For example, NEMA, GBT, TNO, IR, and LMT were not spelled out when first introduced. Additionally, APEX and ASTE were not given their full names. Please review the text to ensure all abbreviations are properly defined on their first mention.

 5. P6. How is the projected detection of asteroids by AtLAST compare with SO.

 6. P6. What is "large collecting area A"?

 7. P7. "for a comparison to SCUBA2" better change to JCMT since in section 4.1 does not mention SCUBA-2.

 8. P7. "fast slewing" how fast it is?

 9. P7. "faster" is italic format.

 10. P8. two "due to" in one sentence in section 2.1 first paragraph.

 11. P9. Figure 1. "5, 25 and 35%" should be "5%, 25% and 35%". In the subplots, the error bars overlap with those in the main plot. Please shift some of them to the left to make all of them visible.

 12. P9. "10's of thousands" should be "tens of thousands".

 13. P9. Footnote for "Small bodies near and far" is needed.

 14. P9. "with ALMA and SCUBA" should change SCUBA to SCUBA-2 or JCMT?

 15. P9. "which corresponds to roughly 1 hour of observation with AtLAST. As discussed above, this is typical for a year of AtLAST observations,". Confused by the exact meaning here, 1 hour to a year.

 16. P10. "3 to 100%" should be "3% to 100%".

 17. P10. what is the diameter for the 6 fields as you already mentioned other 8 circular fields?

 18. P11. ¨At-LAST" should be "AtLAST".

 19. P12. "X-ray binaries (XRB)" can also include extragalactic objects.

 20. P13. Figure 2 should be cited when describe MAXI J1820+070.

 21. P14. "(e.g.)" should be removed.

 22. P14. Figure 3. the subplots cover the frequencies of interest, please point it out.

 23. P15. SVOM has already been launched, please update the information accordingly.

 24. P16. Figure 4, the caption should not describe only the left panel.

 25. P17. Figure 5. the collecting area and integration speed all have the same 69.4 times greater than ACT? Please add citation here to provide the source of this information.

 26. P18. "10s to 100s of square" should be "tens to hundreds of square".

 27. P18. "as many bands a possible" should be "as many bands as possible".

 28. P18. As I do not know the exact FOV, so the estimation of the scanning of a 550 degree^2 region is difficult.

 29. P19. "gammarays" should be "gamma rays".

 30. P19. "A such," is not correct expression.

Is the study design appropriate and does the work have academic merit?

Yes

Is the work clearly and accurately presented and does it cite the current literature?

Yes

If applicable, is the statistical analysis and its interpretation appropriate?

I cannot comment. A qualified statistician is required.

Are all the source data underlying the results available to ensure full reproducibility?

No source data required

Are the conclusions drawn adequately supported by the results?

Yes

Are sufficient details of methods and analysis provided to allow replication by others?

Yes

Reviewer Expertise:

Optical observation of transients. Robotic telescope. Gamma-ray burst.

I confirm that I have read this submission and believe that I have an appropriate level of expertise to confirm that it is of an acceptable scientific standard, however I have significant reservations, as outlined above.

# Reviewer report: Reviewer response for version 1

**DOI:** 10.21956/openreseurope.19113.r43436

**Published:** 2024-08-27

**Authors:** Yuhan Yao

**Affiliations:** 1 University of California Berkeley, Berkeley, California, USA

This article provides a comprehensive overview of the time-domain science goals of the proposed Atacama Large Aperture Submillimeter Telescope (AtLAST). AtLAST is expected to play a crucial role in both conducting surveys in the sub-millimeter band and performing follow-up observations of transients discovered at other wavelengths.

 While I am able to assess the galactic and extragalactic science goals, my familiarity with Solar System science is limited, which may constrain the depth of my feedback in that area.

 === Major comments ===

 It would be beneficial for the authors to provide an estimated timeline for AtLAST, including key milestones such as the start of construction and the expected date of first light.

 Clarification is needed on the operational plan regarding the distribution of time between surveys and follow-up observations. What fraction of telescope time will be dedicated to each?

 Several figures in this article (e.g., Figures 2, 3, and 4) appear to be direct reproductions from previously published papers. Although citations are provided in the figure captions, it is important to confirm whether permission has been obtained for their reuse. Could the authors clarify if such permissions were secured?

 On Page 17, the statement "The submillimeter band is almost the only wavelength range where obscuration is not an issue" might benefit from further elaboration. Could the authors discuss the challenges related to obscuration in the infrared and radio bands?

 The authors are encouraged to provide quantitative estimates for key observational parameters in the summary (Section 5), such as the Field of View (e.g., 1–2 sq deg), sensitivity (e.g., <1 mJy in 1 hour), angular resolution, and slew rate (e.g., ~1 deg per second). Although these details are included in the main text, a summary at the end would be valuable for readers.

 === Minor comments ===

 - The reference for the Zwicky Transient Facility is incorrect. The authors should cite Masci et al. (2019) [Ref 1] and Graham et al. (2019) [Ref 2]

 - On Page 15, the sentence "Observations at X-rays are typically done within minutes, so far with Swift, and in the future with SVOM" should be revised, as SVOM is already conducting scientific observations.

 - On Page 19, the meaning of the sentence "For automated alerting, principal investigators should have an ‘opt-out’ option if their core scientific goals are searches for transients in their fields" is unclear. A rephrasing or further explanation would be helpful.

 - Please double-check the caption of Figure 5. The last line appears to have a formatting issue.

 === Typos ===

 - The reference following "....GRB 990123, followed by a 8.4 GHz radio detection 19 h later" should be in \citep{} instead of \citet{}.

 - Page 18. "As an example of the ‘capability of AtLAST, ..."

 - Page 19. "A such, a great benefit to the community ..."

Is the study design appropriate and does the work have academic merit?

Yes

Is the work clearly and accurately presented and does it cite the current literature?

Yes

If applicable, is the statistical analysis and its interpretation appropriate?

Not applicable

Are all the source data underlying the results available to ensure full reproducibility?

Yes

Are the conclusions drawn adequately supported by the results?

Yes

Are sufficient details of methods and analysis provided to allow replication by others?

Yes

Reviewer Expertise:

time domain astronomy; high energy astrophysics

I confirm that I have read this submission and believe that I have an appropriate level of expertise to confirm that it is of an acceptable scientific standard, however I have significant reservations, as outlined above.

## References

[1] : The Zwicky Transient Facility: Data Processing, Products, and Archive. *Publications of the Astronomical Society of the Pacific* .2019;131(995) : 10.1088/1538-3873/aae8ac 10.1088/1538-3873/aae8ac

[2] : The Zwicky Transient Facility: Science Objectives. *Publications of the Astronomical Society of the Pacific* .2019;131(1001) : 10.1088/1538-3873/ab006c 10.1088/1538-3873/ab006c
